# The MAastricht Instrument for Sustainable Employability – Italian version (MAISE-IT): a validation study

**DOI:** 10.1186/s12889-022-12872-z

**Published:** 2022-03-18

**Authors:** Eleonora Picco, Inge Houkes, Angelique De Rijk, Massimo Miglioretti

**Affiliations:** 1 Department of Psychology, Bicocca Center for Applied Psychology – BiCApP, University of Milano-Bicocca, 20126 Milan, Italy; 2 Department of Social Medicine, Faculty of Health, Medicine and Life Sciences, CAPHRI Care and Public Health Research Institute, Maastricht University, PO Box 616, 6200 MD Maastricht, The Netherlands

**Keywords:** Sustainable employability, Questionnaire, Workers’ well-being, Occupational health psychology, Work-health balance

## Abstract

**Background:**

Governments and employers aim to promote sustainable employability (SE) in aging societies. In the Netherlands, an instrument for capturing the employee perspective on SE, the MAastricht Instrument for Sustainable Employability (MAISE-NL), has recently been developed. This study seeks to validate the Italian version of the MAISE (MAISE-IT).

**Methods:**

The MAISE-IT (a translated and culturally adapted version of the MAISE for the Italian population), the Work-Health Balance questionnaire and a demographic survey (age, gender, education, and occupational activity) were completed online by 455 respondents (328 public administration workers and 127 respondents recruited from social networks). Construct and criterion validity were tested by CFA; reliability, correlational analyses and subgroup differences with ANOVAs.

**Results:**

The CFA analysis revealed that the MAISE-IT consists of 12 scales distributed in four areas: (1) Meaning of SE; (2) Level and Factors affecting SE; (3) Overall responsibility for SE; and (4) Responsibility for factors affecting SE. Construct and criterion validity and reliability were good. Italian workers reported a moderately high level of SE. They regarded employers to be somewhat more responsible for SE than employees.

**Conclusions:**

This study showed the validity of the MAISE-IT in the Italian context. The MAISE-IT is valuable for tapping employees’ needs in order to develop SE interventions tailored to the employee perspective.

**Supplementary Information:**

The online version contains supplementary material available at 10.1186/s12889-022-12872-z.

## Background

European societies and economies are affected by population aging [1]. In view of this demographic change, it is imperative for governments and employers to increase labor market participation and productivity [2]. The employability concept was initially introduced in this context. A recent definition of employability has, in contrast to previous scholars’ definitions and following up previous conceptualizations [3, 4], underlined the importance of making a distinction between employability orientation and employability activities [5]. According to Lo Presti and Pluviano, employability is first a personal mindset that grows over time [5]. As a consequence of this mindset, employability results in several behaviors that aim at developing – for instance – valuable competencies and career networks [5].

Recently, scholars have moved from this individual perspective of employability to focusing on employer responsibilities, adding a time horizon to well-being and health aspects of employability. Accordingly, the concept of sustainable employability (SE) has been introduced. SE refers to the opportunities for employees to function and maintain employment until pension age, preserving their health, vitality, and well-being. According to van der Klink et al. [6], enabling personal and work conditions are both required to gain tangible opportunities for valuable job functioning. In this conception, work offers the chance to contribute to individual and organizational values [6, 7]. However, this definition, based on Sen’s capability approach [8], has been subjected to heavy criticism. Fleuren et al. have underlined how defining SE both as a set of opportunities or capabilities and as the process of converting favorable conditions into SE is quite confusing [9].

More recently, Fleuren et al. [10] have reviewed the conceptualizations of SE existing in literature so far. Among these conceptualizations, an interesting one is those by Le Blanc et al., which is based on the Ability Motivation Opportunity (AMO) framework and defines SE as “the degree to which an employee is willing to carry out his/her current and future work” [11]. Le Blanc et al. have also operationalized SE with three individual indicators – motivation, opportunity and ability to continue working [11]. However, Fleuren et al. have argued that some indicators (e.g., competences) miss in this operationalization, while opportunity to keep working should be considered an antecedent of SE rather than an indicator [10]. Moreover, in SE measurement, Le Blanc et al. use a cross-sectional approach, not addressing the longitudinal component of SE [10]. Hazelzet et al., in their systematic review of SE interventions, have instead proposed that at least four core components should constitute the key indicators of SE: a health component (e.g., work ability and well-being), a productivity component (e.g., performance and turnover), a valuable work component, relying on Sen’s capability approach (e.g., skills and competences), and a long-term perspective component (e.g., future employability), referring to a longitudinal perspective for SE [12].

Fleuren et al. aimed at integrating different SE conceptualizations, coming up with an improved definition [10]. They finally argue SE to mean that “an individual’s ability to function at work and in the labor market, or their ‘employability’, is not negatively, and preferably positively, affected by that individual’s employment over time”. Furthermore, nine measures (i.e., health status, work ability, need for recovery, fatigue, job satisfaction, motivation, employability, skill-gap, and performance) – to be collected at different points of the working life – have been presented as capturing this ability to stay sustainable employable [10]. Importantly, contextual components are not included in this SE conceptualization but considered SE antecedents, and according to Fleuren and colleagues [10] and in agreement with Hazelzet et al. [12] the temporal component of SE is explicitly specified [10]. These reflections have significant implications for how to measure SE.

To promote SE, valid and reliable instruments for assessing employees’ needs and factors that affect SE are needed. Some authors, such as Fleuren et al. [10] refer to combining existing instruments [10, 13–15]. Other authors, in the framework of employability, have developed valid instruments, but mainly based on organization- [3], competency- [4] or individual- centered [16] measures of employability.

**Fig. 1:**
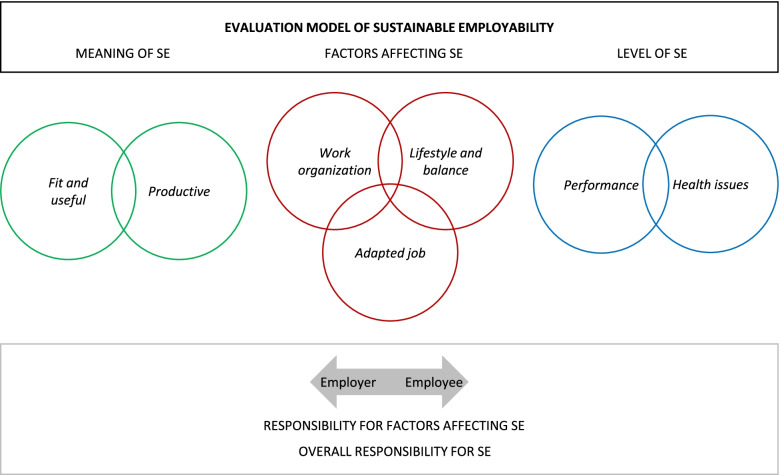
Evaluation model of Sustainable Employability (SE)

In the Netherlands, a new instrument for measuring SE and perceptions of SE from an employees’ perspective has been developed recently: the MAastricht Instrument for Sustainable Employability (MAISE-NL) [17]. This instrument aims at measuring Meaning of SE, Factors affecting SE and Level of SE separately. Factors affecting SE included are: work organizational factors: factors related to job adaptations: and individual factors (lifestyle and balance). For the Factors affecting SE and for SE overall, presumed responsibilities are also measured. Specifically, employees can state how much they think changes in work content, work context, job adaptations, lifestyle and balance and changes to their SE overall to be their responsibility or a responsibility of their employer. Figure 1 represents SE subdimensions, therefore depicting a novel evaluation model of SE.

The MAISE seems to align with the need for SE interventions. Indeed, at present, many SE interventions have been developed, but no firm conclusions regarding their effectiveness can be drawn [7, 12, 18, 19]. In the MAISE, work and individual characteristics have to be modelled in a way that they are associated with SE [10]. Moreover, such characteristics have to interact in a way that personal needs and work demands are reciprocally adjusted [20].

As concerns SE functionings, the MAISE includes the measure of the level of SE, operationalized by two indicators – performance and health impairments (work-induced or not). The importance to the employees of two aspects of sustainable employment, being fit and useful and feel productive, is also assessed. The longitudinal perspective of SE can be covered by using the MAISE repeatedly. The MAISE aims therefore at tapping the employee perspective on factors affecting SE, responsibility for SE and SE itself. These factors are supposed to facilitate employers in better aligning SE interventions with employee needs.

The MAISE-NL has so far been validated in a Dutch working context, and further adaptations in other contexts are needed [17]. It is the purpose of this study to examine the psychometric properties of the version of the MAISE adapted for the Italian population (MAISE-IT) and describe differences between the MAISE-NL and the MAISE-IT. Questionnaire adaptation includes not only translation but also cultural adaptation [20]. To clarify the research setting, we will first describe the Italian context for SE. Because SE is the result of an interplay between various societal and individual levels [21], in doing so, we will take into consideration Italian welfare, legislation, culture, and organizational policies.

### The Italian context of SE

Older Italian workers have traditionally benefited from a high proportion of expenditure on pensions and early retirement ages [22]. To increase the labor force participation of older workers, Minister Fornero’s 2011 reform raised the pension age and modified the mechanism that links pension age and life expectancy [23]. Although the social discourse in Italy is extremely strong, social partners and policy makers have only recently focused on the issue of the employment rate of older workers, and they have not yet addressed sufficiently the questions of working conditions, work ability or well-being at work [22, 24]. As a result, the main sustainable work outcomes for older employees (health, well-being, work-life balance, job security, employability) score among the lowest in the EU [23]. Due to possible health problems, older workers are overall seeing the sustainability of their work at risk, and they are exposed to stereotypes concerning, among other issues, a poor performance [25, 26].

Furthermore, Italy has the highest share of low-skilled adults among the OECD countries; literacy competence among workers is low, and this poses a possible obstacle to increasing wages, well-being, and job satisfaction [27]. The discourse of low educated workers’ SE is overall just starting to emerge in scientific literature [28–31].

Moreover, the limited availability of quality care services for the elderly and for people with disabilities weighs heavily on female sustainable employment and on female sick leave [23]. Women often have to care for sick and aging family members informally; thus, sick leave sometimes provides them with a last resort alternative to take care of family [23]. Even though Italy has a relatively generous long-term leave policy for working caregivers, it does not have a high level of work-time flexibility yet [23]. Globally, very few scientific literature on SE of women has been delivered [32, 33]. Recently, the second welfare paradigm has been the subject of public debate, and new forms of cooperation between the public sector, market and society are in development [27]. The 2016 and 2017 Stability Laws established tax relief for company welfare services. As a result, even with territorial and company dimension disparities, company welfare is growing [27].

In Europe, companies and employers are being increasingly challenged to focus on SE strategies [34]. To develop effective interventions for SE, as it is especially reported in the gray literature, attention to employees’ real needs is required [35]. Therefore, an instrument that fits with Italian employees’ perspectives is needed. Such an instrument is required to spread a culture of SE that – in Italy – is still at the beginning of its development.

### Aim of the study, research questions and hypotheses

This study focuses on the validation of the Italian version of the MAISE and discusses differences between the Italian and Dutch instruments.

The research questions are as follows:

1. What are the validity (construct and concurrent validity) and reliability of the MAISE-IT scales?
2. Are there any subgroup (in terms of gender, age, and education) differences in the MAISE-IT scores?

A priori hypotheses were formulated.

*Hypothesis 1*: We expect to confirm the factorial structure of the MAISE-NL [17] among Italian workers. We also expect the MAISE-IT scales to be correlated with the Work-Health Balance questionnaire (WHBq) scales and index [36]. Finally, we expect Cronbach’s alphas to be equal or higher to 0.60 for all the MAISE-IT scales [37, 38].

*Hypothesis 2a:* We expect women to report a higher job performance than men since they tend to achieve higher levels of organizational capabilities, which may impact their performance [39, 40]. We also expect women to report levels of health issues equal to men since the female gender is negatively associated with a greater proportion of health issues and disabilities, but, in Italy, men tend to be exposed more to various risk factors, including higher smoking rates [41].

*Hypothesis 2b:* We expect older workers to report more health issues than younger workers since age has been found to be negatively associated with health status [42]. We also expect older workers to report an equal job performance than younger workers, since they may at the same time benefit from more training experience in skills that have been reported to be positively associated with job performance [43, 44]. 

*Hypothesis 2c:* We expect lower-educated workers to report worse job performance and more health issues than higher educated employees since education level has been found to be negatively associated with exposure to several work-related risk factors, lower training and chronic diseases [45, 46].

## Method

### Adaptation of the MAISE to develop the MAISE-IT

In the Italian samples, the translated version of the MAISE, the MAISE-IT, was used. Three independent authors translated the items from the English version of the MAISE into Italian, comparing the different resulting versions and producing a final unified Italian version. Then, the authors determined whether the items were pertinent to the Italian working context. Next, a language teacher (not a native English speaker) back-translated the Italian version of the questionnaire into English. Subsequently, both the back-translated and the Italian versions were readapted. A native English speaker and psychology professor then checked for correspondence between the original version of the MAISE, its back-translated version, and the Italian version. Finally, as participants in the questionnaire pilot study expressed some concerns about the meaning of SE, in order to make the concept understandable by a larger sample of participants, a definition of SE based on the literature [6] was added to the Italian version at the beginning of the questionnaire. In this definition, SE referred to opportunities for employees to valuably contribute through their work, safeguarding their health and welfare now and in the future. Opportunities are achieved by means of a facilitating work and personal attitudes and motivations.

### Design

As the main purpose of this study is to establish the factor structure of the MAISE-IT, cross sectional surveys are used to validate the MAISE-IT scales. Two online cross-sectional surveys were conducted in an organizational sample (Public Administration Agency sample, PAA sample) and in a convenience sample (C sample) in Italy.

### Participants and procedure

Between March and April 2018, data were collected in a regional section of an Italian public administration agency (by means of the PAA online survey) involved in environmental protection. The majority of the employees in this regional agency have an intermediate or high level of education. Forty-seven percent of the employees are male and 53% are female. Four hundred thirty seven of the 1,010 employees responded, yielding a response rate of 43.3%. After removing substantially incomplete questionnaires, 328 questionnaires were included in the data analyses. These statistics were very similar to those found for the employee population of the regional public administration agency (56.5% female, 53.2% 40 ≤ age < 55, and 68.0% postsecondary education/university).

In the same period, additional data were collected by means of an online survey (C survey). Respondents were mainly recruited from the northern part of Italy through two social networks (Facebook and LinkedIn). After removing substantially incomplete questionnaires, participants not employed at the time of recruitment, participants with less than 1 year of working experience and participants who were not between 18 and 65 years old, 127 questionnaires were included in the data analyses (68.1% female, 49.5% 18 ≤ age < 40, 70.6% postsecondary education/university and 57% white-collar). To obtain a bigger sample, data obtained from both surveys were aggregated. Table 1 shows descriptive statistics of the PAA sample, the C sample and the final sample (*N* = 455) resulting from data aggregation.

**Table 1:** Sample characteristics: mean age, gender (%), educational level (%), educational level (categories) and occupational activity (%)

Variable	Total Sample	PAA Sample	C Sample
Age (mean)	46.7	48.9	40.2
Gender (%)			
men	40.5	43.5	31.9
women	59.5	56.5	68.1
Educational level (%)			
primary education	0.2	0.3	0
middle education	0.2	0.3	0
lower professional education	5.0	4.6	6.3
secondary education	25.4	26.8	21.4
post-secondary education	3.9	4.0	3.6
bachelor’s degree	7.6	6.2	11.6
master’s degree	42.3	42.8	41.1
post-degree master or PhD	14.9	15.1	14.3
other	0.5	0	1.8
Educational level (categories)			
(1) < post-secondary education	30.9	32.0	27.7
(2) post-secondary education/university	68.7	68.0	70.6
Occupational activity (%)			
white-collars	-	-	57
blue-collars	-	-	9
pink-collars	-	-	13
n.d	-	-	21

*PAA Sample* Public Administration Agency Sample, *C Sample* Convenience Sample

### Measures

#### MAISE-IT

The draft version of the MAISE (MAISE-IT) was used to measure SE in the Italian samples. It consists of 50 items divided over 12 scales and 4 areas. The first area of the MAISE-IT – *Meaning of SE* – consists of 2 scales (10 items): *Fit and useful* (6 items) and *Productive* (4 items). The set begins with “Sustainable employability has the following meaning to me,” and example items for the two scales are “I can do my job without too much stress” and “Being able to do my work until I retire,” respectively. The response scales range from 1, “Strongly disagree,” to 5, “Strongly agree.”

The second area – *Level and Factors affecting SE* – consists of 5 scales (19 items): *Performance* (4 items) and *Health issues* (2 items), *Work organization* (6 items), *Lifestyle and balance* (2 items), and *Adapted job* (5 items). The set *Level of SE* begins with “To what extent do the following statements apply to you?” and example items for the two scales – *Performance* and *Health issues* – are “I have the required knowledge to perform my job” and “My job is stressful,” respectively. The response scales range from 1, “Strongly disagree,” to 5, “Strongly agree.” The set *Factors affecting SE* starts with “Indicate to which extent you believe the following changes could contribute to your sustainable employability.” Example items for the three scales – *Work organization*, *Lifestyle and balance*, and *Adapted job* –, are “Atmosphere improvement within my department/team,” “Start eating healthier” and “Introduce more flexible working hours,” respectively. The response scales range from 1, “Strongly disagree,” to 5, “Strongly agree.”

The third area – *Responsibility for SE* – consists of 1 item: “With whom does the responsibility for sustainable employability lie according to you?” The response scale ranges from 1, “Only with the employer,” to 5, “Only with the employee.”

The fourth area – *Responsibility for factors affecting SE* – consists of 5 scales (18 items): *Lifestyle* (3 items), *Balance* (2 items), *Adapted job* (4 items), *Work content* (4 items), and *Work context* (5 items). The set starts with “Indicate where you feel the responsibility lies for implementing the changes below that would improve your sustainable employability.” Example items for the five scales are “Reach a healthier body weight,” “Find a better balance between my job and private life,” “Introduce more flexible working hours,” “More variation in job activities” and “Improvement of working conditions,” respectively. The response scale ranges from 1, “Only with the employer,” to 5, “Only with the employee.”

#### Demographics

The demographic information collected by the online Italian surveys were gender, age, educational level, and occupational activity (only for the C sample). Educational level was categorized into two categories: (1) < postsecondary education and (2) postsecondary education/university.

#### Correlates of SE

Work-health balance [36] was measured using the Work-Health Balance questionnaire (WHBq). We included work-health balance as a correlate measure as this construct has been considered to be particularly associated with SE functionings, such as job satisfaction [47]. We consider work-health balance (WHB) to be “a state in which the worker feels able to effectively balance health and work needs, considering management’s attention to employee health and the perception of compatibility between one’s personal health situation and job characteristics” [35, p. 376]. The Work-Health Balance questionnaire consists of three factors/scales: Work–health incompatibility (WHI, 6 items), such as “Your job lets you take care of your health”; Health climate (HC, 5 items), such as “In my organization, health prevention involves all levels of the organization”; and External support (ES, 6 items), such as “Your supervisor listens when you talk about your health.” The response scale ranged from 1, “Strongly disagree,” to 5, “Strongly agree.” We used the method described by Gragnano et al. [36] to compute the overall WHB index.

### Data analyses

Construct validity of the MAISE-IT was examined through confirmatory factor analysis (CFA). CFA was performed by means of JAMOVI version 0.9.5.12 [48] which uses the maximum likelihood estimation method. A check of kurtosis and asymmetry values for each of the MAISE-IT scales was conducted a priori. Models were estimated on the basis of the principal component analysis and CFA results for the Dutch MAISE [17]. As SE has to be considered a formative construct, that is a second-order construct with a longitudinal nature, a CFA including all MAISE-IT items, measuring qualitatively different aspects of SE, was not performed as not appropriate to SE model. CFA was instead performed separately for the items measuring SE meaning, SE indicators and level, and responsibility for SE antecedents, as they are supposed to share a conceptual unity [49]. Diverse indices were used to evaluate the fit of the factor structures. The Chi-square index, which should not be significant at *p* < 0.001, was used to assess the exact fit of the model. The comparative fit index (CFI) higher than 0.9, the Tucker-Lewis Index (TLI) higher than 0.9, the standardized root mean squared residual (SRMR) lower than 0.08, and the root mean squared error of approximation (RMSEA) lower than 0.08 were also considered to assess the goodness of fit of the model. Reliability, correlational and comparative (ANOVAs) analyses were performed using IBM SPSS Statistics version 25 [50]. Concurrent validity of the MAISE-IT scales was examined by calculating the Pearson correlations among the MAISE-IT scales and between the MAISE-IT scales and the proxy. Comparative analyses (ANOVAs) were conducted in order to examine subgroup differences in the MAISE-IT scores. Means, standard deviations and 25^th^ and 75^th^ percentiles of the MAISE-IT scales were calculated.

### Ethical measures

Ethical measures are described in the following section. The PAA study was approved by the HR Director. In both the Italian studies, participants were informed of the study by an individual mailing, were free to refuse to participate and were welcomed to ask questions or express concerns about the study. In both Italian studies, the return of a completed questionnaire was taken to imply consent. Data were treated confidentially and anonymously, and the participants’ privacy was guaranteed. The study was part of a larger project on worker well-being, which was approved by the Ethics Committee of the University of Milano-Bicocca (0,025,854/13).

## Results

### Validity of the MAISE-IT

Table 2 presents the results of the CFA (construct validity) of the MAISE-IT items for the Italian sample (areas 1–2 and 4). Factor structure was not tested for area 3 *Responsibility for SE*, as it was measured by means of only 1 item.

**Table 2:** Fit indices of the MAISE-IT areas

		***Chi-2 (df)***	***CFI***	***TLI***	***SRMR***	***RMSEA***
1	Meaning of SE (2 factors)	120 (33)	.932	.907	.041	.076
2	Level and Factors affecting SE (5 factors)	323 (112)	.918	.901	.059	.066
4	Responsibility for factors affecting SE (5 factors)	416 (122)	.932	.915	.006	.073

*CFI* Comparative Fit Index, *TLI* Tucker Lewis Index, *SRMR* Standardized Root Mean Square Residual, *RMSEA* Root Mean Square Error of Approximation

Variable names, labels, factor loadings and Average Variance Extracted are reported in Additional File 1. Factor loadings were all significant at *p* < 0.001.

In the CFA for the MAISE-IT area 1, *Meaning of SE*, the factor structure of the MAISE-NL, consisting of two scales – (1a) Fit and Useful and (1b) Productive – was confirmed. Error terms of two items (SOSD1 and SOSD4) were allowed to correlate in the CFA, since they are related to stress and health issues, respectively, which are both negative aspects of SE.

In the CFA for the MAISE-IT area 2, *Level and Factors affecting SE*, the factor structure of the MAISE-NL, comprising of five scales – (2a) Performance and (2b) Health issues, (2c) Work organization, (2d) Lifestyle and balance and (2e) Adapted job – was not confirmed. It was necessary to delete two items from subscale (2b) Health issues, “I feel that I will be able to do my job until I retire” and “I am rarely absent from work due to sickness”, and two items from subscale (2e), Adapted job, “More attention paid to career paths” and “Change of job tasks/function/activities,” to obtain more acceptable fit indices. Error terms of two items (FATD4 and FATD5) were allowed to correlate in the CFA, as they refer to improvements or adjustments of the daily job.

In the CFA for MAISE-IT area 4, *Responsibility for factors affecting SE*, the factor structure of the MAISE-NL, consisting of five scales – (4a) Lifestyle, (4b) Balance, (4c) Adapted job, (4d) Work content and (4e) Work context – was confirmed. Error terms of three pairs of items (RESD7 and RESD9; RESD10 and RESD14; and RESD17 and RESD18) were allowed to correlate in the CFA since they refer respectively to: working hours, in terms of flexibility or working time; changes related to tasks or activities; and job autonomy, in terms of expansion on the opportunities to apply skills or responsibilities within the job.

Table 3 presents the Pearson correlations of the MAISE-IT scales with the WHB scales and index (criterion validity) as well as the scales’ reliabilities.

**Table 3:** Pearson correlations MAISE-IT scales and items and WHBq scales and index, and Cronbach’s alphas (N ranges from 434 to 455)

# Variable^a^	1a	1b	2a	2b	2c	2d	2e	3	4a	4b	4c	4d	4e	6	7	8	9
***MAISE-IT scales***																	
1a Useful	-																
1b Prod	0.58**	-															
2a Perf	0.16**	0.22**	-														
2b Health	0.11*	0.08	0.00	-													
2c Work org	0.34**	0.25**	-0.01	0.09*	-												
2d Lifestyle	0.09*	.022**	0	0.19**	0.21**	-											
2e Adapted	0.22**	0.18**	0.05	0.35**	0.31**	0.44**	-										
3 Resp. SE	0.07	0.06	0.07	-0.03	-0.03	0	-0.10*	-									
4a Life.-res	0.09	-0.03	0.03	-0.07	0.03	-0.15**	0.12**	0.03	-								
4b Bal.-res	-0.02	0.12*	0.04	-0.07	-0.03	0.04	-0.09*	0.13**	0.05	-							
4c Adap.-res	-0.06	0.11*	-0.01	0.40	0.04	0.21**	0.12**	0.10*	-0.28**	0.39**	-						
4d Content-res	-0.03	0.09	0	-0.01	-0.01	0.17**	0.07	0.14**	-0.18**	0.32**	0.68**	-					
4e Context-res	-0.06	0.12**	0.04	-0.02	-0.01	0.19**	0.06	0.16**	-0.21**	0.50**	0.76**	0.74**	-				
***WHBq scales and index***																	
6 WHI	0.01	0.02	-0.16**	0.59**	0.04	0.22**	0.30**	-0.09*	-0.07	-0.09	0.02	0.02	-0.05	-			
7 HC	0.04	0.07	0.17**	-0.23**	-0.02	-0.01	-0.07	0.17**	-0.01	0.20**	0.19**	0.25**	0.27**	-0.29**	-		
8 ES	0.02	0	0.12**	-0.25**	-0.03	-0.10*	-0.25**	0.11*	0.02	0.10*	0.06	0.11*	0.07	-0.40**	0.47**	-	
9 WHB-i	0.02	0.01	0.20**	-0.53**	-0.04	-0.18**	-0.30**	0.14**	0.06	0.15**	0.07	0.10*	0.15**	-0.86**	0.66**	0.73**	-
Cronbach’s alpha	0.78	0.7	0.75	0.69	0.79	0.91	0.75	-	0.65	0.47	0.86	0.85	0.84	0.87	0.77	0.9	-

^*^p < .05; ** *p* < .01. ^a^Explanation of variable names, *Useful* Fit and Useful, *Prod* Productive, *Per.* Performance, *Health* Health issues, *Work org* Work organization, *Lifestyle* Lifestyle and Balance, *Adapted* Adapted job, *Resp. SE* Overall responsibility for SE, *Life.-res* Responsibility for lifestyle, *Bal.-res* Responsibility for balance, *Adap.-res* Responsibility for adapted job, *Content-res* Responsibility for work content, *Context-res* Responsibility for work context, *WHI* Work–health incompatibility, *HC* Health climate, *ES* External support, *WHB-i* Work-Health Balance index

The reliabilities ranged from acceptable to very good for the majority of the MAISE-IT scales. Reliability was questionable for scale 4b, *Responsibility for balance* (0.47). However, we decided not to modify or delete the scale considering the low number of items (2 items) in this scale.

As one would expect, MAISE-IT scales 2a (Performance) and 2b (Health issues), which both measure the Level of SE, had moderate and significant associations with the WHBq scales, which assess work-health compatibility, health climate and external support at work, and with the overall WHB index. Low to moderate and significant associations were also found between the WHB scales, the index and the MAISE-IT scales 2d (Lifestyle & Balance) and 2e (Adapted job) – which concern Factors affecting SE – and scales 3 (Responsibility for SE) and scales 4b through 4d – which are about responsibility for balance, adapted job, work content and work context. Overall concurrent validity was good. *Hypothesis 1* was, therefore, partially confirmed.

### Subgroup differences in the MAISE-IT scores

Before assessing subgroup differences in the MAISE-IT scores, the means, standard deviations, and 25^th^ and 75^th^ percentiles of the MAISE-IT scales were calculated for the total sample. These are presented in Table 4.

**Table 4:** Means (M), standard deviations (SD), and percentiles of the MAISE-IT scales for the total sample (*n* = 455)

Scale (range 1–5)	#	M	SD	25th percentile	75th percentile
**1. Meaning of SE**					
1a. Fit and Useful	6	4.24	.53	4.00	4.67
1b. Productive	4	3.71	.72	3.25	4.25
**2. Level and Factors affecting SE**					
2a. Performance	4	3.99	.58	3.75	4.50
2b. Health issues	2	2.85	1.00	2.00	3.50
2c. Work organization	6	3.86	.66	3.50	4.33
2d. Lifestyle and Balance	2	2.89	1.19	2.00	4.00
2e. Adapted job	3	3.35	.95	2.67	4.00
**3. Responsibility for employee SE (*n* = 450)**					
Who is responsible for employee SE?	1	2.65	.54	2.00	3.00
**4. Responsibility for factors affecting SE**					
4a. Lifestyle	3	3.86	.75	3.33	4.33
4b. Balance	2	3.28	.74	3.00	3.50
4c. Adapted job	4	2.12	.87	1.50	2.50
4d. Work content	4	2.57	.77	2.00	3.00
4e. Work context	5	2.49	.75	2.00	2.80

A higher score/percentile reflects a more positive score on the particular variable, except for the *Health issues* subscale: here a higher score reflects more health problems. A higher score/percentile on scale 2c-2e means that this particular factor contributes a lot to SE. A higher score/percentile on scales 3 and 4 means that responsibility lies mainly with the employee

According to the Italian respondents, SE first means being fit and useful, referring to the employee perception of having the right knowledge and capacity to perform their job properly, and second, being productive until retirement. According to respondents, the factors affecting SE are work organization and the possibility of adapting their job to their condition and needs more than a healthy lifestyle or a good work-life balance. Respondents regarded SE as a responsibility that lies slightly more with the employer. In particular, they regarded their employer as more responsible for adapting the job to the employee, work context and work content, while they viewed themselves as more responsible for lifestyle and work-life balance.

Table 5 shows gender, age and educational differences tested in the MAISE-IT sample scores.

**Table 5:** Means (M) and standard deviations (SD) of the MAISE-IT scales for the subgroups

**Scale (range 1–5)**	**M (SD)**		**F (df)**	**M (SD)**		**F (df)**	**M (SD)**		**F (df)**
	**Men**	**Women**		** < 55**	**55 + **		**Lower / middle ed. (*n* = 135)**	**Higher ed. (*n* = 300)**	
	**(*n* = 177)**	**(*n* = 260)**		**(*n* = 303)**	**(*n* = 120)**				
**1. Meaning of SE**									
1a. Fit and Useful	4.14 (.58)	4.32 (.49)	11.56 (1)**	4.26 (.53)	4.24 (.56)	.20 (1)	4.25 (.57)	4.25 (.51)	.88 (1)
1b. Productive	3.55 (.74)	3.81 (.69)	14.61 (1)**	3.70 (.72)	3.75 (.74)	.44 (1)	3.82 (.66)	3.66 (.74)	3.69 (1)**
**2. Level and Factors affecting SE**									
2a. Performance	3.91 (.64)	4.03 (.54)	4.62 (1)**	3.99 (.54)	3.94 (.69)	.82 (1)	3.88 (.62)	4.04 (.55)	4.17 (1)**
2b. Health issues	2.74 (1.02)	2.90 (.95)	3.04 (1)*	2.82 (.98)	2.87 (.96)	.30 (1)	2.91 (.98)	2.80 (.97)	1.25 (1)
2c. Work organization	3.72 (.70)	3.94 (.62)	11.76 (1)**	3.93 (.62)	3.65 (.75)	15.66 (1)**	3.74 (.71)	3.91 (.64)	3.27 (1)**
2d. Lifestyle and balance	2.9 -1.15	2.87 (1.22)	.08 (1)	2.92 (1.18)	3.20 (.96)	.07 (1)	3.12 (1.17)	2.77 (1.19)	5.00 (1)**
2e. Adapted job	3.15 (.93)	3.47 (.96)	12.39 (1)**	3.38 (.94)	3.21 (1.00)	2.62 (1)	3.38(1.01)	3.32 (.93)	.19 (1)
**3. Responsibility for employee SE**									
Who is responsible for employee SE	2.59 (.57)	2.68 (.52)	2.77 (1)*	2.65 (.54)	2.64 (.53)	.06 (1)	2.63 (.57)	2.65 (.53)	.09 (1)
**4. Responsibility for factors affecting SE**									
4a. Lifestyle	3.83 (.71)	3.89 (.79)	.52 (1)	3.87 (.78)	3.85 (.72)	.06 (1)	3.84 (.77)	3.88 (.75)	.59 (1)
4b. Balance	3.31 (.77)	3.25 (.73)	.61 (1)	3.29 (.73)	3.32 (.76)	.16 (1)	3.31 (.83)	3.27 (.71)	1.29 (1)
4c. Adapted job	2.16 (.81)	2.07 (.90)	1.07 (1)	2.09 (.84)	2.19 (.94)	1.04 (1)	2.20 (.94)	2.06 (.82)	2.54 (1)
4d. Work content	2.56 (.73)	2.57 (.80)	.02 (1)	2.55 (.77)	2.63 (.78)	.80 (1)	2.63 (.81)	2.53 (.76)	1.03 (1)
4e. Work context	2.50 (.72)	2.48 (.76)	.74 (1)	2.47 (.73)	2.58 (.78)	2.04 (1)	2.56 (.87)	2.45 (.68)	2.00 (1)

^*^ *p* < .10; ** *p* < .05. A higher score reflects a more positive score on the particular variable, except for the Health issues subscale: here a higher score reflects more health problems. A higher score on scales 2c-2e means that this particular factor contributes a lot to SE. A higher score on scales 3 and 4 means that responsibility lies mainly with the employee

Regarding the *Meaning of SE* for employees (area 1), more women than men considered being fit and useful (*F*(df) = 11.56(1), *p* < 0.05) and productive (*F*(df) = 14.61(1), *p* < 0.05) as important factors to be sustainably employable. Additionally, respondents with a low education level considered being productive to be a more important factor than being highly educated (*F*(df) = 3.69(1), *p* < 0.05).

Concerning the description of their own *Level of SE* (area 2), women (*F*(df) = 4.62(1), *p* < 0.05) and highly educated respondents (*F*(df) = 4.17(1), *p* < 0.05) scored higher on Performance, and women (*F*(df) = 3.04(1), *p* < 0.10) reported more health issues. Older respondents did not score significantly differently than younger on Performance (*F*(df) = 0.82(1), *p* > 0.10). *Hypotheses 2a, 2b and 2c* were therefore partially confirmed.

Regarding *Factors affecting SE* (area 2), women (*F*(df) = 11.76(1), *p* < 0.05), younger respondents (*F*(df) = 15.66(1), *p* < 0.05) and highly educated respondents (*F*(df) = 3.27(1), *p* < 0.05) scored higher on Work organization. Women (*F*(df) = 12.39(1), *p* < 0.05) also scored higher on *Adapted job*.

Regarding *Responsibility for SE* (area 3), women regarded themselves as being more responsible than their employer for their SE in general (*F*(df) = 2.77(1), *p* < 0.10). Concerning *Responsibility for factors affecting SE* (area 4), no significant differences in scores were found for gender, age, or educational level.

## Discussion

This study aimed to examine the validity of the Italian version of a new instrument to test aspects of SE: the MAISE-IT. Italian data were collected by means of two online cross-sectional surveys of an organizational and a convenience sample, which were aggregated for the analyses. To our knowledge, this is the first study introducing and evaluating SE in Italy. Not surprisingly, SE knowledge still remains scarce in Italy.

The MAISE-IT’s construct validity, reliability and criterion validity were rather good, partially confirming *Hypothesis 1*. The MAISE-IT seemed well able to capture the different aspects of SE separately: from (responsibility for) SE antecedents to SE meaning and functionings. All scales, except for subscales 2b, *Health issues* and 2c, *Adapted job*, were similar in the Italian and Dutch versions. After considering modification indices, two items from subscale *2b*, *Health issues*, and two items from subscale 2c, *Adapted job*, of the MAISE were not included in the Italian version of the scale: “I feel that I will be able to do my job until I retire” and “I am rarely absent from work due to sickness”, and “More attention paid to career paths” and “Change of job tasks/function/activities”, respectively. These deletions may have been the result of cultural differences between Italy and the Netherlands in the conception of healthy careers that would deserve further deepening. The majority of the MAISE-IT scales’ reliabilities ranged from adequate to very good, except for scale 4b, *Responsibility for balance* (0.47). However, this scale consisted of only a few items (2 items), theoretically related in content.

The MAISE-IT scales had moderate and significant associations with the WHBq scales. In particular, the MAISE-IT scale *Level of SE* was associated with the general WHB index, which refers to factors involved in the process of balancing between health needs and work demands, a process crucial to the quality of working life [47]. The MAISE-IT scale *Health issues* was coherently associated with the WHBq scale *Work-health incompatibility*. The MAISE-IT scale *Performance* was positively associated with the WHB index, the WHB scale *Health climate at work* and the WHB scale *External support received at work for health*. However, the MAISE-IT scale *Performance* was negatively associated with the WHB scale *Work-health incompatibility*. This seemed reasonable, as more health issues have a negative impact on work performance [49]. To summarize, the sustainable employability concept was moderately associated with the WHB concept without overlapping it.

Means, standard deviations and percentiles for all MAISE-IT scales for the Italian samples are provided in this paper. Italian respondents considered SE to primarily mean being fit and useful, and secondly, being productive until retirement. This perception is in line with a general need – due to changes to the work ecosystem –, for reskilling, developing adaptive capabilities, and for having the right knowledge and capacity to perform the job properly till pension age [51, 52]. In general, Italian employees considered their SE to be moderately high (approximately 4 on the productive scale, with a range of 1–5 and almost 3 on the health issues scale). Remarkably, Italian employees, similar to Dutch ones [17], regarded employers as more responsible for their work content, work context and job adaptations and themselves as more responsible for their lifestyle. Nevertheless, the majority of SE interventions still focus on health and lifestyle [24, 53], with the risk of not meeting employees’ needs. In our sample, work-life balance was mainly considered a shared responsibility rather than an employer’s responsibility, as in the Netherlands. In Italy, a high level of work-time flexibility among employees is indeed still required from employers [23].

Scores on the various scales of the MAISE-IT varied between subgroups. In particular, younger employees – more so than older employees – considered work organization to be a factor affecting SE. Adequate SE actions targeting younger workers seem, therefore, to be required, as they might have less autonomy to manage their working life [23, 54]. Women in our research group – more so than men – scored higher on performance and considered being fit and useful as well as being productive as important meanings of SE. They considered SE to be affected by job adaptations but themselves as being more responsible than the employer for their general SE. Women tend to achieve higher levels of capabilities, which could have an impact on their productivity [39, 40]. However, women are often forced to organize their work differently than they would prefer [45] in terms of work-time arrangements, occupational choices and pay [55]. Because of the traditional Italian female role of caring for the family, much effort to balance work and family life is still required of women [23, 55]. Gender-specific interventions are therefore recommended [24]. Less educated employees – more so than highly educated employees – considered SE to mean being productive and reported a worse job performance (*Hypothesis 2b*). While this situation is plausible, as less educated Italian workers generally experience a very low training intensity, which is accompanied by lower productivity [45, 56, 57]. Again, dedicated attention to vulnerable groups is required so that all can receive the opportunities to be sustainably employable. Overall, the Italian version of the MAISE seems to be as capable as the Dutch version to detect subgroup differences related to specific perspectives on SE and division of responsibility for SE [17].

Considering the MAISE-IT and MAISE-NL mean scores [17], in remarkable contrast to Dutch employees, Italian employees in our samples regarded overall SE to be a responsibility that lies somewhat more with the employer. Italian employees ask employers to deal more with questions pertaining to employee working conditions [27] and to take on more responsibility for SE. As old welfare models are being overhauled and companies are increasingly asked to cooperate with the public sector and society, Italian employers are, accordingly, starting to provide more comprehensive welfare services (e.g., supplementary health care, supplementary pension funds, work-life balance strategies, etc.) [27]. Dutch employers have a long tradition of bearing responsibility for health promotion and age management at work due to legislative improvements aimed at reducing long-term sickness absence [58–60]. Income provision, rehabilitation, and reintegration have indeed legally moved from social insurance providers to employers. Employers are therefore responsible for short- and long-term income and for organizing return-to-work services [58, 60]. Thus, Dutch employees already feel SE to be a responsibility shared with their employer. Many Dutch employers, particularly in larger organizations, already pay attention to SE, although there is a huge variety in the types of interventions delivered on a corporate level [60, 61].

Second, Italian employees reported worse job performance but fewer health problems than Dutch employees. A more inclusive education system could indeed, among northern countries, result in better work outcomes [45]. The Netherlands is one of the countries that has had the biggest share of adults who rate their health to be good [62]. It is therefore not easy to explain differences in reported health problems. However, measures of self-reported health may be affected by social and cultural factors, which make cross-country differences in perceived health status difficult to interpret [62].

### Methodological limitations

The sample in this study included employees of varying ages, genders, educational levels, and occupational activities. However, some issues have to be addressed with regard to the generalizability of the results. No data were available on the representativity of the public administration agency sample for the public administration sector at a regional or national level. Respondents belonged to only one work sector, and the sample size might be too restricted to extend results to a general working population. The convenience sample was relatively small as well and not representative of the general working population. Two slightly different samples were also aggregated for the analyses.

The response rate was not high (43.3%), perhaps because the questionnaire was rather long. Illiterate employees or employees not able to read Italian sufficiently would have been missed; it is unknown how large this group is. A participant selection bias could have consistently affected the online data collection. The aggregated sample consisted mainly of highly educated and healthy people, and women were slightly overrepresented, although there was variety with regard to age.

As a limitation, error terms of some items from different subdimensions were allowed to correlate in the CFA. As scale 3, *Responsibility for overall SE*, consists of only 1 item, no factorial analysis could be performed. One scale – *Health issues* – consisted of only 2 items, advocating for approaching separately some relevant questions such as absenteeism. The reliability of scale *Responsibility for balance*, consisting also of 2 items, was slightly low, advising for the inclusion of additional items in improved versions of the questionnaire. More or different factors than those included in the MAISE-IT could be considered as affecting SE. For instance, not only personal and contextual factors may be considered as SE antecedents, but also the balance between the two could be included in the SE antecedents’ measurement. Moreover, SE functionings could be related to other key outcomes of a sustainable career, such as happiness, satisfaction, or to (perceived) employability [63]. As this was a cross-sectional study, the longitudinal nature of SE was not tested. Finally, the construct validity was tested by means of only one SE correlate. Different or more concepts could be considered as SE correlates.

### Implications for future research and practice

Further validation of the MAISE-IT in larger samples from various work sectors and among vulnerable groups, such as employees with health issues, older employees, self-employed employees, and less educated employees, is recommended. It is also recommended to replicate the questionnaire validation within a large representative group of employees in Italy. As small and medium-sized companies comprise the majority of the Italian productive panorama [27] it is suggested to specifically validate the MAISE-IT among these firms. To target workers at all levels of education, a further translated version of the simplified MAISE-NL version for lower educated employees should be developed and validated [64], and/or help with assessment (e.g., via telephonic administration) should be provided.

Test–retest reliability studies, studies among younger employees, and longitudinal studies are recommended as well to improve the SE of each employee later in life and to monitor long-lasting processes. As the MAISE-IT separately measures factors affecting SE and SE itself, repeated use of the instrument can capture the longitudinal nature of SE by evaluating the long-term effects of SE interventions [9, 17]. SE measures can, therefore, be repeated at multiple time points throughout the employee working life, to monitor SE level over time. In doing so, a particular attention should be dedicated to understanding the role of gender and education level in the process of SE capabilities development.

Specific interventions, policy regulations, and campaigns should be developed to make younger groups of employees more aware of opportunities to increase their SE. Furthermore, effective interventions for SE should consider cultural aspects. Differences can indeed be found in values and individual preferences at the level of national cultures and in practices at the level of organizational cultures [65]. As employees in different contexts might have different ideas about SE, an instrument that measures perspectives on different SE dimensions is required [17].

In Italy, efforts to sensitize employers to SE interventions already exist, but new policies and laws are needed. If SE is considered a shared responsibility, government and social partners are called to play an essential role in terms of policy development. Specifically, SE interventions should mainly focus on work organization and fitting the employee perspective.

## Conclusions

The MAISE-IT can be used to measure the meaning and level of SE from employees’ perspective. Employees’ ideas about factors affecting SE and responsibility for SE can be measured as well. These measures will facilitate the employers’ and policymakers’ decisions for choosing and developing group- or subgroup-level SE interventions. Effectiveness evaluation of the interventions, by using the MAISE-IT, can then follow.

## Supplementary Information

**Additional file 1.**

## Data Availability

The datasets generated during and/or analyzed during the current study are not publicly available due to the privacy of the participants, but are available from the corresponding author on reasonable request.
