# Supplementary material for: The MAastricht Instrument for Sustainable Employability – Italian version (MAISE-IT): a validation study
Source: BMC Public Health. 2022 Mar 18;22:541. doi: 10.1186/s12889-022-12872-z (PMC8933982; doi:10.1186/s12889-022-12872-z)
Supplement: Supplementary file 1 — Additional file 1. [file 12889_2022_12872_MOESM1_ESM.docx]

**Supplementary table 1.** Variable names, labels, factor loadings and Average Variance Extracted.

| Variable | Subscale | | Name | English label | Standard. factor loading | Average Variance Extracted |
| --- | --- | --- | --- | --- | --- | --- |
| 1 - Meaning of SE | |  |  |  |  |  |
|  | 1a - Fit and Useful | |  |  |  | .378 |
|  |  |  | SOSD1 | I can do my job without too much stress | .393 |  |
|  |  |  | SOSD2 | I have the right knowledge to perform my job well | .702 |  |
|  |  |  | SOSD3 | I enjoy my job | .626 |  |
|  |  |  | SOSD4 | I do not develop physical health issues as a result of my job | .572 |  |
|  |  |  | SOSD5 | The capacity to do my job efficiently | .755 |  |
|  |  |  | SOSD6 | The feeling of performing useful activities | .574 |  |
|  | 1b - Productive | |  |  |  | .396 |
|  |  |  | SOSD7 | Being able to do my work until I retire | .583 |  |
|  |  |  | SOSD8 | Try to keep my absenteeism limited | .711 |  |
|  |  |  | SOSD9 | I can make money | .480 |  |
|  |  |  | SOSD10 | I am productive while working | .713 |  |
| 2 - Level and Factors affecting SE | |  |  |  |  |  |
|  | 2a - Performance | |  |  |  | .472 |
|  |  |  | LOSD1 | I have the required knowledge to perform my job well | .525 |  |
|  |  |  | LOSD3 | I am efficient at my job | .845 |  |
|  |  |  | LOSD4 | I feel that my job activities are useful | .493 |  |
|  |  |  | LOSD6 | I am productive while working | .810 |  |
|  | 2b - Health issues | |  |  |  | .552 |
|  |  |  | LOSD7 | My job is stressful | .831 |  |
|  |  |  | LOSD8 | I have work-related physical health issues | .643 |  |
|  | 2c - Work organization | |  |  |  | .377 |
|  |  |  | FATD1 | Atmosphere improvement within my department/team | .451 |  |
|  |  |  | FATD3 | Expansion of education/development possibilities | .642 |  |
|  |  |  | FATD4 | More variation in job activities | .480 |  |
|  |  |  | FATD5 | More challenging job activities | .679 |  |
|  |  |  | FATD6 | To receive more appreciation for the job that I do | .652 |  |
|  |  |  | FATD7 | The chance to apply my knowledge / skillset to my job better | .728 |  |
|  | 2d - Lifestyle and Balance | |  |  |  | .831 |
|  |  |  | FATD10 | Reach a healthier body weight | .876 |  |
|  |  |  | FATD11 | Start eating more healthy | .946 |  |
|  | 2e - Adapted job | |  |  |  | .483 |
|  |  |  | FATD14 | Decrease in job pressure | .785 |  |
|  |  |  | FATD15 | Introduce more flexible working hours | .668 |  |
|  |  |  | FATD17 | Reducing weekly working hours | .622 |  |
| 3 - Responsibility for overall employee SE | | | |  |  |  |
|  |  |  | RES | With whom does the responsibility for improving employee’s sustainable employability lie, according to you? |  |  |
| 4 - Responsibility for factors affecting SE | | | |  |  |  |
|  | 4a - Lifestyle | |  |  |  | .514 |
|  |  |  | RESD1 | Move more | .260 |  |
|  |  |  | RESD2 | Reach a healthier body weight | .877 |  |
|  |  |  | RESD3 | Start eating more healthy | .840 |  |
|  | 4b - Balance | |  |  |  | .366 |
|  |  |  | RESD4 | Find a better balance between my job and private life | .750 |  |
|  |  |  | RESD5 | Learn to deal with stress better | .411 |  |
|  | 4c - Adapted job | |  |  |  | .583 |
|  |  |  | RESD7 | Introduce more flexible working hours | .801 |  |
|  |  |  | RESD8 | More attention paid to career paths | .865 |  |
|  |  |  | RESD9 | Reducing weekly working hours | .737 |  |
|  |  |  | RESD10 | Change of job tasks / function / activities | .631 |  |
|  | 4d - Work content | |  |  |  | .574 |
|  |  |  | RESD14 | More variation in job activities | .792 |  |
|  |  |  | RESD15 | More challenging job activities | .895 |  |
|  |  |  | RESD17 | The chance to apply my knowledge / skillset to my job better | .705 |  |
|  |  |  | RESD18 | Obtain more responsibility within my job | .610 |  |
|  | 4e - Work context | |  |  |  | .513 |
|  |  |  | RESD6 | Decrease in job pressure | .651 |  |
|  |  |  | RESD11 | Atmosphere improvement within my department / team | .512 |  |
|  |  |  | RESD12 | Improvement of working conditions | .737 |  |
|  |  |  | RESD13 | Expansion of education / development possibilities | .840 |  |
|  |  |  | RESD16 | To receive more appreciation for the job that I do | .794 |  |
